# Context-dependent lexical ambiguity resolution: MEG evidence for the time-course of activity in left inferior frontal gyrus and posterior middle temporal gyrus

**DOI:** 10.1016/j.bandl.2018.01.001

**Published:** 2018

**Authors:** Giovanna Mollo, Elizabeth Jefferies, Piers Cornelissen, Silvia P. Gennari

**Affiliations:** a University of York, Department of Psychology, UK; b Northumbria University, Department of Psychology, UK

## Abstract

An MEG study investigated the role of context in semantic interpretation by examining the comprehension of ambiguous words in contexts leading to different interpretations. We compared high-ambiguity words in minimally different contexts (*to bowl, the bowl*) to low-ambiguity counterparts (*the tray, to flog*). Whole brain beamforming revealed the engagement of left inferior frontal gyrus (LIFG) and posterior middle temporal gyrus (LPMTG). Points of interest analyses showed that both these sites showed a stronger response to verb-contexts by 200 ms post-stimulus and displayed overlapping ambiguity effects that were sustained from 300 ms onwards. The effect of context was stronger for high-ambiguity words than for low-ambiguity words at several different time points, including within the first 100 ms post-stimulus. Unlike LIFG, LPMTG also showed stronger responses to verb than noun contexts in low-ambiguity trials. We argue that different functional roles previously attributed to LIFG and LPMTG are in fact played out at different periods during processing.

## Highlights

• The time course of lexical ambiguity resolution was examined with MEG.
• High-ambiguity words disambiguated by minimal contexts (to bowl vs. the bowl) were compared to low-ambiguity controls.
• LIFG and LPMTG showed early (∼100 ms) context-by-ambiguity interactions, suggesting controlled retrieval.
• Both sites showed later interactions and ambiguity effects, suggesting a role in disambiguation.
• Distinct functions previously attributed to these regions play out at different time points.

## 1 Introduction

One of the most important properties of natural languages is that word meanings are flexibly and dynamically computed as a function of context. Most English words in isolation have multiple meanings (e.g., *watch*) and require contextual information to cue the appropriate interpretation. Even the meaning of a seemingly unambiguous word such as *piano* can activate different features depending on context, e.g., *moving the piano* vs. *playing the piano* (Tabossi, 1988). Thus, word meanings are dynamically computed each time a word is encountered using different sources of information (prior knowledge, context) to converge on an interpretation. This aspect of language is fundamental as it ultimately allows speakers to convey multiple meanings and describe multiple real and imagined situations with a finite number of words.

Here, we used MEG to investigate the brain mechanisms implementing such context-dependent interpretation processes, and in particular, their temporal dynamics. To capture basic processes that would otherwise be overshadowed by complex sentential stimuli, we used minimally different two-word visual stimuli (presented simultaneously). The stimuli contained ambiguous words that can be used as either nouns or verbs with equivalent frequency, e.g., *bowl* or *hammer*, and are thus syntactically and semantically ambiguous, i.e., their word class and meaning (e.g., object or action) are not specified in the lexicon. These ambiguous words therefore require contextual information to arrive at the correct interpretation as an object or action. Because these alternative interpretations are clearly disambiguated by minimal functional contexts such as *the* or *to,* phrases such as *to bowl* provide a unique opportunity to examine the effect of functional context in interpreting the same ambiguous word (Gennari, MacDonald, Postle, & Seidenberg, 2007). We therefore compared the comprehension of phrases containing high-ambiguity words, e.g., *the bowl, to bowl,* with phrases containing low-ambiguity words that are most frequently encountered with the same interpretation, e.g., *the tray*, *to dig* (see Table 1). The comparison between high-ambiguity and low-ambiguity phrases reveals the processes that are differentially engaged in lexical ambiguity resolution, whereas interactions between contexts and ambiguity—the main focus of our analyses—indicate the contrasting effect of context for each ambiguity condition.

**Table 1:** Examples of stimulus phrases in each condition.

	High-ambiguity Word	Low-ambiguity Word
Noun context	*the bowl*	*the tray*
	*the sling*	*the leash*
	*the brush*	*the blade*
	*the hook*	*the pliers*
	*the handcuff*	*the hatchet*
	*the fork*	*the rod*
	*the skewer*	*the chisel*
	*the rake*	*the spade*
	*the ring*	*the hoop*
	*the clip*	*the jug*
	*the stick*	*the rope*

Verb context	*to bowl*	*to dig*
	*to sling*	*to knead*
	*to brush*	*to untie*
	*to hook*	*to sift*
	*to handcuff*	*to fasten*
	*to fork*	*to flog*
	*to skewer*	*to unlock*
	*to rake*	*to slay*
	*to ring*	*to bind*
	*to clip*	*to pluck*
	*to stick*	*to wipe*

Ambiguity resolution has been extensively investigated in psycholinguistics and cognitive neuroscience of language (Duffy et al., 1988, MacDonald et al., 1994, Mason and Just, 2007, Rodd et al., 2012, Rodd et al., 2010, Simpson, 1984). Many of these studies have examined the role of meaning frequency, e.g., dominant vs. subordinate meanings, as well as the role of sentential or discourse contexts in biasing towards one or another interpretation, either before or after the ambiguous words is encountered. Comprehenders may revise or reanalyse an initial dominant interpretation in favour of a subordinate one, if information indicates the need to do so (e.g., the mention of a river in the context of *bank,* for which the dominant interpretation is the institution). These processes not only require sentence composition, and sometimes discourse-level processes, but also contextually-elicited priming or revision processes in working memory before or after the ambiguous word is encountered. In the present work, we aimed to isolate lexical disambiguation by a minimal functional word context presented simultaneously with the ambiguous words, thus avoiding sentential composition or subsequent revision processes involved in accessing an ultimately incorrect meaning.

Prior research with equi-biased ambiguous words such as those used here has shown that these words initially activate semantic features consistent with their alternative interpretations, even in disambiguating contexts such as *I bought a watch*. For example, the word *watch* in *I bought a watch* primes words related to either of the two competing meanings (e.g., *look, clock)* immediately after word presentation. However, as the stimulus onset asynchrony increases to 200 ms or later, priming only obtains for the context-relevant interpretation (e.g., *clock*) (Seidenberg, Tanenhaus, Leiman, & Bienkowski, 1982). These results suggest that equally frequent meanings are initially activated, and that only later context leads to the correct interpretation. In the electrophysiological literature, effects of context on responses measured at the lexical word are observed around 200–250 ms after word presentation and continue to play a role until around 400 or 500 ms (Federmeier et al., 2000, Ihara et al., 2007, Lee and Federmeier, 2006). This pattern was found by Lee and Federmeier (2006), who used minimal phrases like *to duck* or *the duck* as in the present study. ERP effects around 400 ms (N400 component) have been strongly associated with semantic interpretation and integration of word meanings with prior context (Kutas & Van Petten, 1994). Less clear is what early P200 and frontal negativity effects may indicate, as predictions and expectations from the experimental setting and sentential contexts may also play a role (Lewis, Wang, & Bastiaansen, 2015). Taken together, these findings suggest that when understanding equi-biased words, many interpretations are immediately activated due to the equally strong associations between a word form and its meanings, while by 200 ms, context narrows the range of interpretations.

Previous imaging research has also demonstrated that the left inferior frontal gyrus (LIFG) and the left posterior middle temporal gyrus (LPMTG) are critically involved in ambiguity resolution and context-dependent interpretation (Bedny et al., 2007, Chan et al., 2004, Hagoort, 1993, Noonan et al., 2013, Rodd et al., 2012), and are furthermore functionally and anatomically connected (Catani et al., 2002, Davey et al., 2016, Glasser and Rilling, 2008, Hallam et al., 2016, Rilling et al., 2008, Saur et al., 2008). In particular, an fMRI study using the present stimuli indicated that both LIFG and LPMTG were both modulated by ambiguity and context (Gennari et al., 2007). High-ambiguity phrases elicited more activity than low-ambiguity phrases in LIFG and LPMTG, and *to-*contexts elicited more activity than *the-*contexts. Importantly, high-ambiguity phrases containing the same word (e.g., *to bowl* vs. *the bowl*) also elicited more activity in *to*-contexts than *the*-contexts in these regions, suggesting that more processing resources are recruited when computing action meanings as a function of context. This is consistent with multiple findings reporting that morpho-syntactically marked verbs engage LIFG and LPMTG more strongly than nouns, likely due to the verbs’ multiple semantic event-based features and syntactic role in sentences (Perani et al., 1999, Shapiro et al., 2005, Thompson-Schill et al., 2005, Tyler et al., 2004, Vigliocco et al., 2011). Therefore, it is argued that the interplay between these regions implements context-dependent interpretation and ambiguity resolution.

However, previous fMRI results indicating co-activation of LIFG and LPMTG do not provide sufficient temporal and spatial resolution to investigate in detail the role and specific contribution of these regions to word interpretation in minimal functional contexts. The indeterminacy inherent in fMRI data is clearly exemplified by different views that have been put forward concerning the role of these regions. For example, LIFG (also referred to as ventrolateral prefrontal cortex and including BA44 and 45) has been alternatively proposed to perform (a) top-down allocation of attention or controlled retrieval of task-relevant features that would not automatically be activated in a bottom-up fashion, e.g., attending to word letters or specific semantic features according to task instructions (Badre and Wagner, 2002, Sakai and Passingham, 2006, Wagner et al., 2001), (b) selection between competing semantic alternatives following initial automatic activation of multiple meanings, some of which may be task-irrelevant, and thus, need to be inhibited (Badre et al., 2005, Badre and Wagner, 2002, Janssen, 2012, Ralph et al., 2017a, Thompson-Schill et al., 1997, Thompson-Schill et al., 2005). Although some studies have suggested that mid-LIFG, where functional peaks for control-demanding semantic tasks are often observed, shows effects of *both* controlled retrieval and selection (Badre et al., 2005, Davey et al., 2016, Noonan et al., 2013), these processes might still be separated in time.

**Fig. 1:**
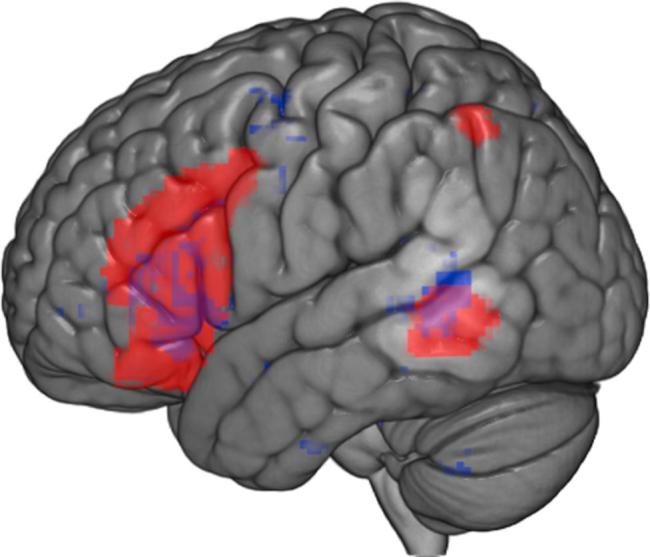
The brain regions in red show the semantic control network reported in the meta-analysis of Noonan et al., 2013. The verb and action knowledge map in blue are the results of an automated meta-analysis using Neurosynth.org (Yarkoni, Poldrack, Nichols, Van Essen, & Wager, 2011). This map shows the activation reported in 110 fMRI studies archived on the database, using “verb” as searching term. Purple shows regions in LIFG and pMTG where these meta-analyses for verb processing and semantic control overlap. The overlap in pMTG has been revealed more prominently by clipping off the cortex to reveal activation within the gyri. (For interpretation of the references to colour in this figure legend, the reader is referred to the web version of this article.)

The role of LPMTG is perhaps even more controversial. On the one hand, LPMTG has been proposed to store and supply semantic information and lexical features pertaining to actions and events (Martin & Chao, 2001). Research consistent with this view has shown that LPMTG responds more strongly to verbs than nouns, to animate events compared to inanimate ones, and to objects with strong action associations, compared to other object types (Beauchamp et al., 2004, Bedny et al., 2008, Humphreys et al., 2013, Kable et al., 2005, Kable et al., 2002, Tranel et al., 2003). This view is also consistent with language processing models arguing that the temporal lobe supplies lexical meaning to unification and control processes taking place in prefrontal cortex (Hagoort, 2005, Hagoort, 2014, Hickok and Poeppel, 2007). On the other hand, LPMTG has been argued to support controlled semantic retrieval together with LIFG. This view is supported by fMRI studies showing that LPMTG responds to context-dependent interpretations and controlled retrieval, along with LIFG (Badre et al., 2005, Davey et al., 2015, Davey et al., 2016, Gennari et al., 2007, Noonan et al., 2013, Whitney et al., 2011). This view is also supported by inhibitory transcranial magnetic stimulation studies showing disruption of controlled retrieval (e.g., retrieval of non-automatic semantic features) when stimulation is applied to both sites (Davey et al., 2015, Jefferies, 2013, Whitney et al., 2011). Thus, while it is clear that LIFG and LPMTG are part of the semantic and conceptual retrieval network, LPMTG shows similar functions to those of LIFG, rather than simply supplying semantic information. The ambiguity inherent in the role of LPMTG is illustrated in Fig. 1, which shows that brain regions implicated in semantic control (from the meta-analysis of Noonan et al., 2013) overlap with regions linked to verb and action knowledge. This common response to verbs/actions and tasks requiring semantic control might occur because both of these situations involve constraining conceptual retrieval to suit a context (Davey et al., 2015, Davey et al., 2016).

While both LIFG and LPMTG co-activate in control-demanding semantic tasks and during the comprehension of actions and verbs, it is possible that they contribute to distinct processes such as controlled retrieval and semantic selection *at different time points*—information that fMRI is not well suited to reveal. At an early-stage, controlled retrieval involves setting up a semantic context to guide later processing – enabling later retrieval to be focussed on currently-relevant but weak meaning features. Either LIFG or LPMTG might support this aspect of controlled retrieval within the first 200 ms (since the studies reviewed above suggest that, after around 200–250 ms, context-dependent semantic features for equi-biased ambiguous words are selectively accessed). We expect that this early effect of establishing a context will be most marked for ambiguous words in verb contexts compared to noun contexts (*to bowl* vs. *the bowl*), because action features tend to require more demanding retrieval, as discussed above. Moreover, we expect that early effects of establishing a context will involve stronger responses to high-ambiguity phrases compared to low ambiguity ones, because unlike lexically-specified action and object meanings, sensitivity to action vs. object interpretations cannot occur without the context. These two predictions entail the possibility of early context by ambiguity interactions, according to which the effect of context would be larger for high-ambiguity than low-ambiguity phrases. Such results in LPMTG would be inconsistent with a view of this site as simply supplying action semantic features in a bottom-up fashion. At subsequent stages of processing, effects of ambiguity in LIFG and LPMTG might then indicate a role for these regions in *selecting* contextually-appropriate interpretations, as suggested by context integration effects in the N400 and the selection account of LIFG. Thus, if LIFG and LPMTG are involved in ambiguity-resolution taking account of the functional context, we would also expect both these regions to show ambiguity effects and interactions with context at a later stage, since the verb > noun context effect should be increased for high-ambiguity words if contextual information is used to resolve ambiguity at these sites.

To examine these possibilities, we conducted a reading comprehension study using magnetoencephalography to examine the oscillatory dynamics of phrase comprehension over time, containing high-ambiguity and low-ambiguity words preceded by *the* or *to* (Table 1). The two words in a phrase (e.g., *to bowl*) were presented simultaneously, rather than in sequence as in previous ERP studies, so simultaneous influences of context and ambiguity could be observed early on. This design is well suited to establish links with previous imaging results and to examine oscillatory activity in response to the phrases as a whole, as pursued below, but it is less well suited to establish explicit comparisons with previous ERP results. To examine the overall pattern of behavioural responses outside the scanner, we pre-tested the stimuli with an identical design to that of the MEG study. In both studies, participants were asked to read phrases for meaning in such a way that they could answer subsequent comprehension questions referring to the action or object interpretation.

## 2 Methods

### 2.1 Participants

15 participants were tested in the behavioral pre-test study and 21 participants (11 females and 10 males) in the MEG study. All participants were students at the University of York, native English speakers, and with no known neurological disorder. All participants provided written consent before taking part in the study. The behavioural study was approved by the Ethics Committee in the Psychology Department (University of York), whereas the MEG study was approved by the Ethics Committee at the York Neuroimaging Centre. Five participants were excluded from MEG data analysis because more than 60% of the trials had to be discarded during the artefact rejection procedure (see below for details).

### 2.2 Materials

The same materials used in Gennari et al., 2007 were used in this experiment. 40 high-ambiguity words were matched on an item-by-item bases for use frequency and character length with 40 low-ambiguity nouns and 40 low-ambiguity verbs that had dominant uses as noun and verb respectively (see Table 1). The high-ambiguity words were equi-biased, i.e., they had similarly frequent object and action interpretations (or noun and verb uses) in English. These words were thus not only semantically ambiguous but also word-class ambiguous. We used the Bank of English/Cobuild corpus (Sinclair, 1995), which contains 200 million words and is annotated according to noun and verb uses, to extract the total frequency for each use. The log_10_ transforms of these frequencies were used for frequency matching. The mean log_10_ frequency for noun and verb uses of high-ambiguity words was 3.24 and 3.19 respectively and there was no significant difference between these frequencies. Most matching low-ambiguity words only have one attested use in the corpus, except for 19 words (out of 80) that had a low frequency alternative (the mean log frequency difference between the high and low frequency alternative of these cases was 1.16). The full list of stimulus words can be accessed at http://www-users.york.ac.uk/~spg500/stim.pdf. The mean log frequency of low-ambiguity words was 3.22 for nouns and 3.27 for verbs. Comparisons of the log frequencies across the high-ambiguity and low-ambiguity words were not significant (t < 1). The same was true for comparisons of word length (see Table 2). One half of the high-ambiguity words had related meanings in their noun and verb uses as in *hammer*, where the action implies the object. The other half had unrelated meanings or sometimes both related and unrelated ones, as in *clip*. This relatedness grouping was not investigated in this study due to low statistical power for this contrast, as in the previous fMRI study (Gennari et al., 2007).

**Table 2:** Mean log frequencies and word length across conditions.

Conditions	Log-frequency	ST	Word length	ST
High-ambiguity noun uses	3.24	0.48	4.95	1.30
High-ambiguity verb uses	3.18	0.50	4.95	1.30
Low-ambiguity nouns	3.22	0.48	5.18	1.08
Low-ambiguity verbs	3.27	0.42	5.18	1.47

Because number of senses can also affect the richness of the semantic representation activated (Rodd, Gaskell, & Marslen-Wilson, 2002), we computed the number of senses for each stimulus word according to the senses listed in two dictionaries (Encarta World English Dictionary, New Oxford American Dictionary). The mean number of senses for high-ambiguity words was 7.8, including both object and action senses, whereas low-ambiguity words such as *chisel, pliers, knit* and *squirt* had an average of 2.78 senses per word. Among high-ambiguity words, noun uses had an average of 4 senses, whereas verb uses had an average of 3.8 senses. Thus, our high-ambiguity words were ambiguous in many ways. However, although it is possible that sense ambiguity played a role initially in the experiment, the minimal context in which they were presented (*to* and *the)* did not provide information to access a particular sense, nor did the questions throughout the experiment. Therefore, it is likely the participants quickly learned during the experiment that only object vs. action distinctions were going to be referred to. Moreover, although we do not have information about the frequency of these senses, we judged that most high-ambiguity words had a dominant sense, and frequency of the interpretation is the relevant factor for automatic activations.

### 2.3 Experimental design

In both the pre-test study examining the stimulus comprehension times and the MEG study, high-ambiguity words were presented in both noun and verb phrasal contexts (*the-* and *to*-contexts). Each participant saw all stimulus conditions and the entire stimulus set (160 phrases total). To this set, we added 20 additional low-ambiguity words to increase the likelihood of low-ambiguity words and thus reduce the probability of expecting ambiguous words, but these words were not included in the analyses. The order of presentation was counterbalanced across subjects by rotating the first and second half of the stimulus list. Moreover, within each half of the experimental list, the order of the high-ambiguity phrases was also counterbalanced, i.e., half of the high-ambiguity words appeared in a noun context first and the other half in a verb context first. These constraints therefore controlled for word repetition effects across different contexts. Except for these ordering constraints, all items were randomly assigned a location in the stimulus list. There were a total of 8 different stimulus orderings presented across participants. Comprehension questions were randomly inserted after a stimulus phrase in 46 trials to guarantee that participants read the phrases for meaning. The questions referred to properties of objects such as their typical uses, physical characteristics, properties of the event referred to by the verb phrases or short dictionary definitions. Examples are given in Table 3. Half of the questions were false and half were true. Considering the whole stimulus set (including the additional 20 low-ambiguity words), a question appeared on the screen on average every 3.96 trials, ranging from 2 to 7 trials. Thus, participants could not predict when a question would appear after reading a phrase, which aimed to keep their focus on meaning throughout the experiment.

**Table 3:** Example of questions used in the experiment.

Stimulus phrase	Question	Expected response
the bolt	part of locks?	yes
the hammer	has a handle?	yes
the broom	for cleaning?	yes
to saw	involves a tool?	yes
to sew	done to fabric?	yes
the jewel	an ornament?	yes
to buckle	to fasten?	yes
the ladder	a car part?	no
to knead	done to enemies?	no
the brush	for fishing?	no
to reel	to listen?	no
to shovel	to telephone?	no
to bowl	with a tool?	no
the sling	type of furniture?	no

Finally, to increase statistical power—which would be difficult to achieve otherwise due to the highly specific nature of the stimuli and the noisy nature of MEG data (we have indeed rejected between 9% and 40% of trials in our data set due to artifacts)—we repeated the presentation of the stimulus lists described above. This meant that some facilitation effects might occur the second time a phrase was processed, i.e., priming across the first and second stimulus blocks. Moreover, facilitation might also occur across presentations of ambiguous words, which were repeated in a list, albeit in different contexts and with different interpretations. To address these issues, we conducted a pre-test of the stimuli in a behavioral task. This allowed us to evaluate whether interaction effects would still obtain despite priming and in particular, whether averaging across blocks was justified, as we planned to do in the MEG data analyses.

### 2.4 Stimulus pre-test study

The pre-test study requested overt responses to the stimuli to determine the average pattern of reading times under identical conditions as those used in the MEG study. In this pre-test, each trial started with the presentation of a phrase that remained on the screen until the participants pressed a button box (middle button) indicating that they have finished understanding the meaning of the phrase. These button presses provided the measure of reading time. After the stimulus phrase was presented, either another stimulus phrase or a question would appear (see above). Stimulus phrases were presented in large 40pt white letters in black background, whereas questions were presented in red letters companied by a question mark. This cued participants to provide a response on a right or left button of the box, in which YES and NO responses were labelled. Participants were instructed to read the phrases for meaning with the aim of answering subsequent comprehension questions if prompted to do so. Participants were also instructed to keep the middle finger of their dominant hand on the middle button of the box to minimise hand movements. Inter-trial times were randomly varied between 1500 ms and 3000 ms to minimise expectations due to periodicity. Before the experiment, participants practiced the task and saw examples of the type of questions they would be asked.

All response times (RT) to experimental phrases up to 5000 ms were included. Analyses did not include RTs to questions or the additional 20 low-ambiguity phrases described in Section 2.1. Because individuals varied greatly in their means (some were faster readers than others), we computed z-scores for each participant and excluded values that fell more that 3.5 standard deviations from each condition’s mean z-score. These exclusions represented less than 1% of the whole data set.

#### 2.4.1 Results of stimulus pre-test

Accuracy in comprehension questions was 87% correct on average, suggesting that participants paid attention to meaning. There was no difference in correct responses to meaning questions across conditions (mean high-ambiguity noun phrases: 88%, mean high-ambiguity verb phrases: 88%, mean low-ambiguity nouns: 87%, mean low-ambiguity verbs: 86%). This suggests that ambiguity did not influence the responses to the questions.

A repeated-measures ANOVA with ambiguity (high vs. low) and functional context (noun vs. verb) and block (first and second list presentations) as repeated factors and mean RT per participant as dependent variable revealed a main effect of block (F(1,14) = 11.87, p = .004), no effect of phrasal context (F(1,14) = 1.11, p = .31), no effect of ambiguity (F(1,14) = 0.53, p = .48), an interaction between ambiguity and context (F(1,14) = 7.07, p = .02), and an interaction between block and context (F(1,14) = 9.09, p = .009). Overall, in the second block, RTs were 158 ms faster than in the first block, indicating repetition priming (main effect of block). The interaction between block and functional context obtained because verb-contexts benefited more from repetition than noun-contexts. No other interactions were observed. Importantly, there was no three-way interaction, suggesting that the critical interaction between context and ambiguity was not influenced by stimulus repetition. The interaction between ambiguity and context also obtained in each block when analysed separately (block 1: F(1,14) = 4.45, p = .05; block 2: F(1,14) = 12.67, p = .003) (see Table 4). This suggests a similar pattern of results across blocks, despite repetitions.

**Table 4:** Mean Response times (in milliseconds) in the stimulus pre-test study.

		Noun context	verb context
		Mean (SD)	Mean (SD)
Block 1	High-ambiguity	866 (483)	888 (515)
	Low-ambiguity	885 (488)	818 (425)

Block 2	High-ambiguity	676 (349)	721 (373)
	Low-ambiguity	714 (382)	708 (377)

Average	High-ambiguity	769 (409)	804 (440)
	Low-ambiguity	799 (426)	763 (392)

Since we are interested in the pattern of results that would obtain by averaging across the two presentation blocks to mimic the averaging of our subsequent MEG study, we computed a repeated-measures ANOVA on the average RTs obtained for each participant irrespective of presentation block. As in the previous ANOVA, there was an interaction between phrase context and ambiguity (F(1,14) = 7.48, p = .02) and no main effects (see Table 4). Separate pair-wise t-tests were conducted to examine the nature of this interaction. It was found that high-ambiguity words in verb contexts were read more slowly than high-ambiguity words in noun contexts (t(1,14) = 2.40, p = .03) and low-ambiguity verb phrases (t(1,14) = 2.70, p = .02). There was also an advantage for low-ambiguity verb contexts compared to low-ambiguity noun contexts (e.g., *to sharpen* vs. *the spade*), which was also present in the first block, where these phrases were seen for the first time (t(1,14) = 2.26, p = .04). This suggests that the functional context helped the interpretation of lexical verbs more than lexical nouns. Taken together, the results of the pre-test study indicate that the interaction between ambiguity and context obtains across and within presentation blocks despite priming effects: high-ambiguity verb contexts resulted in more processing difficulty than low-ambiguity verb contexts, whereas the opposite was true for high- and low-ambiguity noun contexts.

### 2.5 MEG study

#### 2.5.1 Procedure

Using the same stimulus lists described in the experimental design above, all participants in the MEG study saw all stimulus conditions twice. Stimulus phrases were presented in large 40pt white letters in black background for two seconds. After this, a cross would appear on the centre of the screen until the next stimulus or question was shown. Inter-trial times (between stimuli or questions) were randomly varied between 1500 ms and 3000 ms. As in the pre-test study questions were presented in red letters companied by a question mark until the participant press a button on a box where YES or NO responses were labelled. The same instructions as in the pre-test study were used. Thus, the only difference between the pre-test study and the MEG study was the presentation of the stimulus phrases, which remained on the screen for two seconds, instead of eliciting an overt response.

#### 2.5.2 Data acquisition and pre-processing

Participants were seated in a dimly lit magnetically shielded room. MEG data were collected at a sample rate of 678.17 Hz and pass-band filtered between 1 and 200 Hz, using a whole-head 248-channel system, Magnes 3600 (4D Neuroimaging, San Diego, California), with the magnetometers arranged in a helmet shaped array. MEG signals were segmented into epochs of 1300 ms length, starting 500 ms before the target onset. Epochs were visually inspected and manually rejected when contaminated by eye blinks, movement artefacts or electrical noise. Statistical analyses included only datasets with at least 60% of trials. We did not record electrooculography (EOG). On average, 20% of the trials were rejected from these datasets (min 9% - max 40%). Before the experiment, participants’ head shape and the location of five head coils were recorded with a 3-D digitizer (Fastrak Polhemus). In a separate session, anatomical MRI images were acquired with a GE 3.0 T Signa Excite HDx system (General Electric, USA), using an 8-channel head coil and a sagittal-isotropic 3-D fast spoiled gradient-recalled sequence. During data processing, each participant’s structural MRI image, the digitized coils positions and head shape were co-registered using a surface-matching technique adapted from (Kozinska, Carducci, & Nowinski, 2001) to constrain source localization.

#### 2.5.3 Beamforming analysis

The spatial and temporal resolution of the MEG recordings was exploited in a two-step analysis: first, we examined the response of the whole brain to the task (collapsing across conditions) at a coarse frequency resolution and in a broad time range. This stage of the analysis provided an unbiased way of identifying sites important for the task across conditions. Secondly, we examined points of interest (POIs) in frontal and temporal lobe sites that were strongly engaged by the task and that fell within areas previously identified through fMRI meta-analyses as being relevant for both semantic control and verb/action understanding (see Fig. 1). At these points of interest (POIs), we examined responses at a finer frequency and temporal resolution, to consider differences between experimental conditions. Since earlier studies of language and semantic processing have found that differences between experimental conditions tend to be reflected in changes in oscillatory power at specific times and frequencies, whole-brain beamforming which aggregates data across many frequencies or multiple time points is unlikely to be sensitive to our experimental manipulations (Klein et al., 2014, Mollo et al., 2017).

For both source-space analyses, neural sources were reconstructed using a modified version of the vectorised, linearly-constrained minimum-variance (LCMV) beamformer described by Van Veen, van Drongelen, Yuchtman, and Suzuki (1997) and referred by Huang et al. (2004) as Type I beamformer, implemented in the Neuroimaging Analysis Framework pipeline (NAF, York Neuroimaging Centre), using a multiple spheres head model (Huang, Mosher, & Leahy, 1999). An MEG beamformer (spatial filter) estimates the signal coming from a location of interest while attenuating the signal coming from other points in the brain. This is achieved by constructing the neuronal signal at a given point in the brain as the weighted sum of the signals recorded by the MEG sensors. Independent beamformers were reconstructed for each point in the brain, in each of three orthogonal directions separately. In our analysis, the covariance matrix used to generate the weights of each beamformer was regularized using an estimate of noise covariance as described in Prendergast, Johnson, Hymers, Woods, and Green (2011) and Hymers, Prendergast, Johnson, and Green (2010). This procedure was performed separately for each frequency and condition and/or analysis window, in order to maximize sensitivity to the effects of interest (Brookes et al., 2008, Brookes et al., 2011). The outputs of the three spatial filters at each point in the brain (referred to as a Virtual Electrode or “VE”) were summed to generate the total oscillatory power. For the whole-brain analysis, a noise-normalized volumetric map of source total power was produced over a given temporal window and within pre-specified frequency bands. For the region of interest analysis, the time course information at the location specified was reconstructed and the time-frequency decomposition was computed using Stockwell Transforms (Stockwell, Mansinha, & Lowe, 1996). The analysis strategy and the parameters used for the current study were similar to those used in recent MEG studies of visual word recognition and object naming (Klein et al., 2014, Mollo et al., 2017, Urooj et al., 2014, Wheat et al., 2010).

##### 2.5.3.1 Time-frequency analysis: whole brain

This analysis aimed to characterize the response of the brain to the task as a whole to inform the selection of POIs for more detailed investigation. The oscillatory activity through the cortex was estimated separately in four broad frequency bands (5–15 Hz, 15–25 Hz, 25–35 Hz and 35–50 Hz), comparing a baseline period of 200 ms before stimulus onset (passive period) with total power across all experimental conditions during several post-stimulus intervals that were all 200 ms long (e.g., 0–200 ms; 200–400 ms; 400–600 ms post-stimulus onset).

**Fig. 2:**
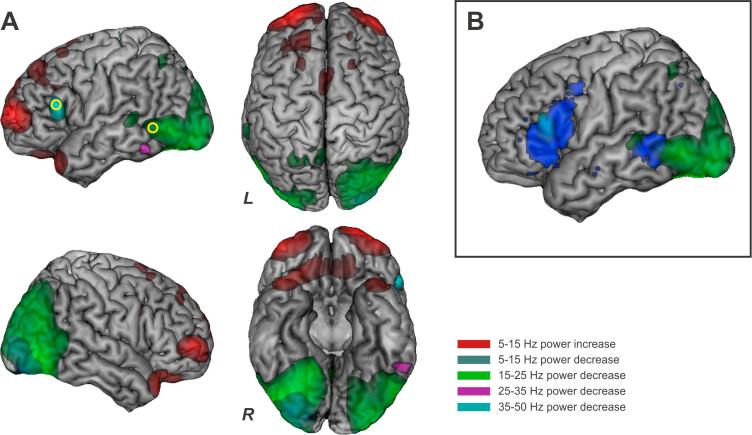
(A) Three-dimensional rendered images of the neuronal responses at 200–400 ms during the task performance compared to pre-trial baseline (p = .01 corrected); different colours refer to different frequency bands and power changes: power increases at 5–15 Hz are represented in red. The remaining colours indicate power decreases at 5–15 Hz in blue (overlaid in the picture with green), at 35–50 Hz in cyan, at 25–35 Hz in purple and at 15–25 Hz in green. POIs are shown as yellow circles. (B) The POIs selected for the time-frequency analysis fall within regions implicated in both semantic control and verb processing identified by fMRI meta-analyses. The blue map represents the brain regions in common between the semantic retrieval network and the verb/action knowledge map presented in . The clusters in LIFG and LPMTG reported in the blue map overlap with the neural responses observed in whole brain beamforming analysis at 35–50 Hz and 15–25 Hz. (For interpretation of the references to colour in this figure legend, the reader is referred to the web version of this article.)

These frequency bands represent a subdivision of the frequency spectrum in steps of 10 Hz (or 15 Hz in the case of the gamma band), and roughly match the frequencies of alpha, low and high beta and low gamma bands, although their purpose was simply to characterise strong sources of oscillatory power across the whole brain in general terms, to support the selection of POIs for the second step of analysis in which we could examine responses across the full range of frequencies across conditions. The post-stimulus 200–400 ms window is displayed in Fig. 2 because this period was the first to show significant peak activity in areas overlapping with our areas of interests (LIFG, LPMTG, Fig. 2), as described below. The baseline window of 0–200 ms was the same length as the active windows, and reflected a compromise between obtaining reasonable low frequency resolution and the need to avoid edge effects in the analysis (since the epoch only began 500 ms before the target was presented). This window length should make it possible to resolve frequencies down to 5 Hz.

A cubic lattice of point sources was defined within the brain with 5 mm spacing and an independent set of beamformers were used to compute the neural activity index at each point of the grid. For each point, a paired-sample t-statistic was computed between active and passive windows at each frequency band, generating separate t-maps for each participant. Individual participant's t-maps (which were initially co-registered with their individual brain scans) were then transformed into standard space and superimposed on the MNI template brain with the cerebellum removed using MRIcroN software (www.mricro.com).

In order to determine whether the difference between active and passive periods was statistically significant for each point on the lattice, we built up a null distribution by randomly relabelling the two time points for each participant and each voxel, using the permutation procedure developed by Holmes, Blair, Watson, and Ford (1996). We established the maximum t-value obtained with random relabelling across 10,000 permutations. We then compared the real distribution of t-values in our data with the maximum t-value obtained from the permuted data (relabelling the active and passive windows). Maximum statistics can be used to overcome the issue of multiple comparisons in neuroimaging analyses (i.e. controlling experiment-wise type I error (Holmes et al., 1996)), since the approach uses the highest permuted t value across the brain to provide a statistical threshold for the whole lattice of points, over which the null hypothesis can be rejected (Nichols & Holmes, 2004). The whole brain beamforming results in Fig. 2A show those voxels in the brain that have t-values equal or higher than the top 1% t-values present in the null distribution.

##### 2.5.3.2 Time-frequency analysis: points of interest

For the points of interest (POI) analyses, the LCMV beamformer approach was used to reconstruct the source activity at two main points of interests (or virtual electrodes; VEs) in the left hemisphere, in LIFG and LPMTG. Within the broad areas shown in Fig. 1 previously defined by prior meta-analyses of semantic control and verb processing (Noonan et al., 2013), we identified the peaks of maximum activation across all conditions in the whole-brain analysis shown in Fig. 2A and B. These peaks in oscillatory power were taken from different broad frequency bands – whichever generated the strongest signals within the region of interest. The left inferior frontal gyrus (LIFG) coordinate was defined using the peak response within the 35–50 Hz band (MNI coordinates *x* = −56, *y* = 22, *z* = 18). This location was within a few millimetres of previously reported sites implicated in this task and in semantic control more widely in mid-ventrolateral prefrontal cortex (Badre et al., 2005, Gennari et al., 2007). The posterior middle temporal gyrus (LPMTG) coordinate was within a region showing a significant response in the 15–25 Hz band (MNI coordinates *x* = −58, *y* = −50, *z* = −6). This site corresponded to the peak reported in previous meta-analyses (Noonan et al., 2013), which was also within a few millimetres of other previously reported sites (Davey et al., 2015, Gennari et al., 2007). We also examined an additional site in left anterior superior temporal gyrus (LASTG), within the anterior temporal lobe, which is reported in Supplemental Materials (see below). Although this site is not implicated in semantic control or verb processing and was therefore expected to show a different pattern of results from LIFG and pMTG, it is strongly linked to verbal semantic tasks (Murphy et al., 2017, Ralph et al., 2017b, Visser et al., 2012) and has been implicated in combinatorial semantics in MEG studies (Bemis and Pylkkänen, 2013, Westerlund and Pylkkänen, 2014). The site we selected corresponded to a region showing a task-induced power increase from 5 to 15 Hz in whole-brain beamforming (MNI coordinates *x* = −44, *y* = 24, *z* = −28).

The time-series of each POI was reconstructed by means of separate beamformers (Huang et al., 2004). Stockwell transforms (Stockwell et al., 1996) were used to compute time-frequency plots for each participant in each condition over a time window from −500 to 800 ms and a frequency range from 5 to 50 Hz (frequency resolution 1.33 Hz). Within this time window, we examined a post-stimulus interval from 0 ms (stimulus onset) to 600 ms, and normalised the power per frequency bin with respect to mean power in a baseline period prior to stimulus presentation (−250 to −50 ms). The VE data were extracted beyond the time windows used in this analysis to avoid artefacts linked to edge effects. Our examination of task effects for 600 ms post stimulus onset captures the time period where prior ERP and MEG effects have been reported (Lee and Federmeier, 2006, Mollo et al., 2017). Although semantic processing is likely to be more extended in time, eye movements and blinks increase beyond 600 ms. This interval also roughly agrees with the average processing time in our pre-test study (793 ms) once motor response preparation is excluded, which is estimated to last between 100 and 150 ms (Leuthold, Sommer, & Ulrich, 1996).

The Stockwell transform, implemented in the NAF software, uses a variable analysing window length, which is automatically adapted along the frequency range according to the sample rate and the trial length. We examined total power, which includes both the phase-locked and non-phase locked components of the signal (Hillebrand & Barnes, 2005). The advantage of examining total power is that this signal captures changes in oscillatory power that are not phase-locked to an event (i.e., that are generated at slightly different time points across trials and participants). This is important because these so-called “induced” responses are perhaps likely to play a role in aspects of semantic processing that are focussed on the interpretation and integration of meanings with a context, and have already been shown to play a key role in reading and visual word recognition tasks (Cornelissen et al., 2009, Pammer et al., 2004, Wheat et al., 2010).

To compare the time frequency representations between experimental conditions, we computed generalized linear mixed models (GLMM) using PROC MIXED in SAS (SAS Institute Inc., North Carolina, US). This type of statistical analysis, unlike permutations, allows for more flexible modelling of the data. Time-frequency plots of percentage signal change between conditions were treated as two dimensional arrays of small time-frequency tiles, indexed in the model by three main effects, each of which is defined as a class variable: time, frequency and the interaction between time and frequency. Therefore, a repeated measures factor was included in each GLMM to account for the fact that each participant’s time-frequency plot is made up of multiple time-frequency tiles. We also controlled for time-frequency (or spatial) co-variance in the spectrogram by assuming the estimates of power followed a Gaussian distribution: consequently a Gaussian link function was used in the model. The time-frequency (spatial) variability was integrated into the model by specifying an exponential spatial correlation model for the model residuals (Littell, Milliken, Stroup, Wolfinger, & Schabenberger, 2006). Finally, the data were resampled at a frequency resolution of 2 Hz and time resolution of 25 ms, the smallest time and frequency bin consistent with model convergence. This time-frequency resolution proved optimal in other similar published studies (Klein et al., 2014, Urooj et al., 2014, Wheat et al., 2010). The most important outcome from the statistical modelling was to identify where in the spectrograms percentage signal change was statistically significantly different from zero. To do this, we computed the predicted population margins from the GLMMs and compared them using tests for simple effects by partitioning the interaction effects, controlling for multiple comparisons. The statistical contours on the spectrograms encompass time-frequency tiles fulfilling both of the following criteria: (a) the difference between conditions reached *p* < .05; (b) any region in the time-frequency plot defined by (a) also showed a response that was significantly different from zero in at least one of the two contributing conditions.

## 3 MEG results

Whole brain responses to the task overall were computed separately for four frequency bands by averaging across the experimental conditions. This analysis compared the oscillatory activity during the active period between 200 and 400 ms after the presentation of the stimulus phrase with a ‘passive’ period (−200–0 ms) during which a fixation cross was present on the screen, Fig. 2A.

Anterior and frontal brain regions showed a significant power increase at 5–15 Hz (in red) and a significant decrease in power in the gamma band (35–50 Hz, cyan). The latter was localized to the motor strip and left frontal operculum. Neuronal activity in posterior regions displayed a significant decrease in power localized over the right occipital pole/cerebellum at 5–15 Hz (blue) and left inferior temporal cortex at 25–35 Hz (purple), along with a wider involvement of the temporal-parietal-occipital cortices bilaterally at 15–25 Hz (green). This pattern is consistent with previous MEG studies showing low frequency increases in total power and decreases in power, relative to a resting baseline, in visual, temporal and frontal regions such as LIFG (Cornelissen et al., 2009, Dale et al., 2000, Urooj et al., 2014). A straightforward interpretation of these power decreases is that they reflect an increase in desynchronised neural activity relative to oscillatory activity at rest (see below): such responses have been shown to correlate with task-related BOLD responses in fMRI (Hanslmayr et al., 2016, Hanslmayr et al., 2012, Hanslmayr et al., 2011, Singh et al., 2002).

### 3.1 Point of interest analyses

The time course of activity for points-of-interest was reconstructed in the range of 5–50 Hz to examine the power changes in the frequency domain over time, across conditions. The section below describes effects for LIFG and LPMTG, while Supplementary materials show results for LASTG.

**Fig. 3:**
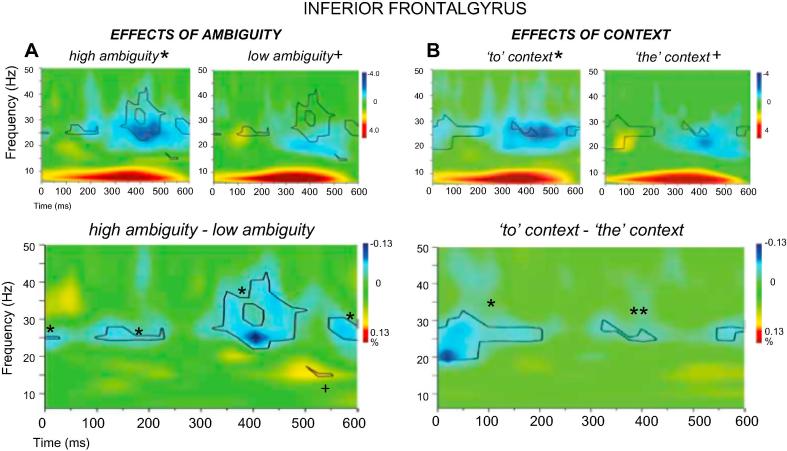
Total power changes and comparisons between conditions for (A) high and low-ambiguity phrases and (B) verb phrases (*to* context) and noun phrases (*the* context) within LIFG. The top panels in (A) and (B) show total power changes for each experimental condition. Orange-red and blue-dark blue colours in the time-frequency plots indicate significant power increase or decrease compared to a passive baseline period, respectively. The lower panels in (A) and (B) show the differences between the two conditions, with black lines enclosing regions that are statistically significant. These between-condition differences are also shown in the top panels to further qualify the nature of these contrasts. To aid interpretability, crosses and asterisks in the contrast plots represent areas that can be unambiguously attributed to a larger power change relative to baseline in a particular condition (e.g., * = *to* context > *the* context, or high-ambiguity > low-ambiguity). (For interpretation of the references to colour in this figure legend, the reader is referred to the web version of this article.)

**Fig. 4:**
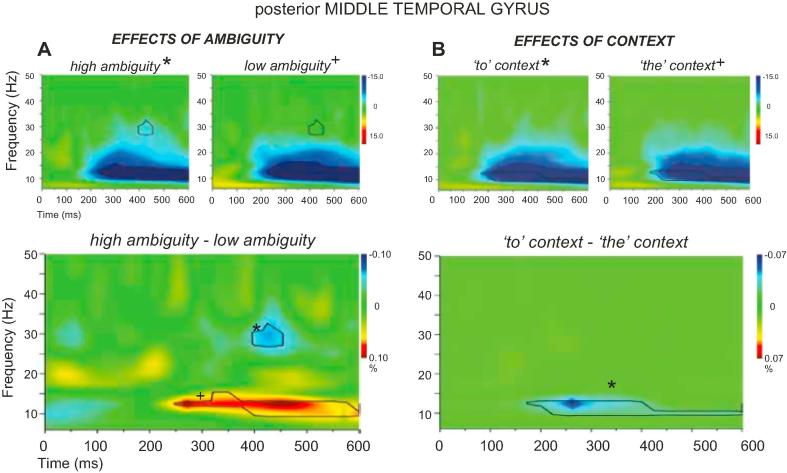
Total power changes and comparisons between conditions for (A) high and low-ambiguity phrases and (B) verb phrases (*to* context) and noun phrases (*the* context) within LPMTG. The top panels in (A) and (B) show total power changes for each experimental condition. Orange-red and blue-dark blue colours in the time-frequency plots indicate significant power increase or decrease compared to a passive baseline period, respectively. The lower panels in (A) and (B) show the differences between the two conditions, with black lines enclosing regions that are statistically significant. These between-condition differences are also shown in the top panels to further qualify the nature of these contrasts. To aid interpretability, crosses and asterisks in the contrast plots represent areas that can be unambiguously attributed to a larger power change relative to baseline in a particular condition (e.g., * = *to* context >*the* context, or high-ambiguity > low-ambiguity). (For interpretation of the references to colour in this figure legend, the reader is referred to the web version of this article.)

Within LIFG and LPMTG, we computed the main effect of ambiguity (high-ambiguity vs. low-ambiguity phrases), the main effect of context (e.g., *the* vs. *to* phrases), and the interaction between these two factors (by comparing the effect of context for high-ambiguity words and low-ambiguity words separately and comparing the difference of these differences). We expected the effect of context to be increased for ambiguous words, since on these trials, context provides critical information to disambiguate meaning. Fig. 3, Fig. 4 show the main effects in time-frequency plots for LIFG and LPMTG respectively. Effects of ambiguity are shown in the top panel (Fig. 3, Fig. 4A) while effects of context are shown in the lower panel (Fig. 3, Fig. 4B). In both cases, the first row of the panel shows the response to each condition compared to the pre-stimulus passive period, while the second row shows contrasts between conditions.

Total power changes in response to a stimulus can either reflect increases or decreases in oscillatory power relative to a resting baseline. In line with many studies in the literature using MEG to investigate language and memory tasks, we observed power increases at relatively low frequencies (e.g., in theta), particularly in the prefrontal site, and then decreases in total power in response to the presentation of a stimulus in beta and low gamma frequencies across conditions, up to around 50 Hz, at both sites (Lam et al., 2016, Urooj et al., 2014). These task-evoked *decreases* in total power are thought to reflect an *increase* in neural activity that is not phase-locked across trials, and allows the efficient representation and processing of information (Hanslmayr et al., 2016, Hanslmayr et al., 2012). As a consequence, an increased engagement of a region in one condition relative to another may give rise to a stronger response characterized by either positive values (shown in red) or negative values (shown in blue) in the total power plots. To aid interpretability, crosses and asterisks in the contrast plots represent areas that can be unambiguously attributed to a larger power change relative to baseline in a particular condition (e.g., ∗ = *to* context > *the* context, or high-ambiguity > low-ambiguity).

#### 3.1.1 Main effect of ambiguity: High-ambiguity vs. low-ambiguity phrases

Fig. 3A and 4A show the time-frequency plots for high-ambiguity vs. low-ambiguity trials, for LIFG and LPMTG respectively. In LIFG, all phrase types compared to baseline elicited strong and sustained increases in oscillatory power in the theta range (around 6–10 Hz). Increases in theta at frontal sources have been previously linked to memory encoding/retrieval and working memory load (Klimesch, 1999, Nunez et al., 2001, Ward, 2003) – thus, this effect might reflect sustained internal attention and retrieval from memory across conditions. LPMTG did not show this event-related increase in low-frequency power.

Compared to baseline, both sites showed event-related decreases in power across conditions at higher frequencies: these were most marked in beta and low gamma (15–40 Hz) in LIFG, and in alpha and beta frequencies (8–25 Hz) in LPMTG. The strength and extent of this response was found to vary across conditions. LIFG showed consistent effects of ambiguity (i.e., bigger event-related power decreases for high-ambiguity > low-ambiguity items), extending across the epoch and peaking at around 400 ms. The strong LIFG response for high-ambiguity trials was focused on the middle of the beta band (25 Hz). In contrast, LPMTG showed stronger and more sustained event-related decreases in power when there was no lexical ambiguity (i.e., differences between conditions at this site were primarily low-ambiguity > high-ambiguity). The response to high-ambiguity and low-ambiguity trials was similar in shape, peaking in the alpha band (8–15 Hz) across conditions and extending from 250 ms post-stimulus to the end of the epoch. However, high-ambiguity trials did elicit stronger event-related power decreases than low-ambiguity trials at around 30 Hz and 400 ms post-stimulus onset in LPMTG.

These results are consistent with the hypothesized role of LIFG in ambiguity resolution. LIFG showed a strong and sustained response to ambiguity, which commenced before 200 ms of stimulus onset and was maintained to the end of the epoch: thus this region may support controlled retrieval and semantic selection processes that take time to complete. LPMTG largely showed the opposite pattern – i.e., a stronger response to low-ambiguity items – consistent with its hypothesized role in semantic retrieval, which may be weakened when the interpretation is unclear. Nevertheless, LPMTG did show an ambiguity effect at 400 ms post-stimulus suggesting that this site might also participate in ambiguity resolution.

#### 3.1.2 Main effect of context: verb contexts vs. noun contexts

This contrast examines differences between action vs. object interpretations irrespective of whether the stimulus phrase contained a word that needed disambiguation (e.g., *to bowl/to dig* vs. *the bowl/the tray*), and in this respect it does not necessarily capture the role of context in disambiguation. Nevertheless, we reasoned that if LIFG and/or LPMTG play a role in controlled retrieval, i.e., in detecting circumstances in which retrieval must be constrained to suit the linguistic context, we would expect an early response to verb over noun phrases. This is because the function word *to* specifies that semantic retrieval must be constrained in order to focus on action/verb features, which engage these brain areas and are more costly to retrieve (Shapiro and Caramazza, 2003, Shapiro et al., 2001, Vigliocco et al., 2011). Fig. 3B and 4B show the time-frequency plots for verb vs. noun contexts, for LIFG and LPMTG respectively. LIFG showed early event-related power decreases before 200 ms at 20–30 Hz in response to verb phrases compared to noun phrases, and similar power decreases at 300–400 ms and 550 ms. These effects overlapped in frequency and time with the effects of ambiguity, suggesting this site might play a role in controlled retrieval or in the focusing of attention on specific aspects of the stimuli such as the context function word. In contrast, LPMTG showed a sustained power decrease at 10–20 Hz from 200 ms onwards in response to verb phrases, which overlapped in frequency and time with the greater response to *low-ambiguity* items. This is consistent with a contribution of LPMTG to action interpretations.

#### 3.1.3 Interactions between ambiguity and context

**Fig. 5:**
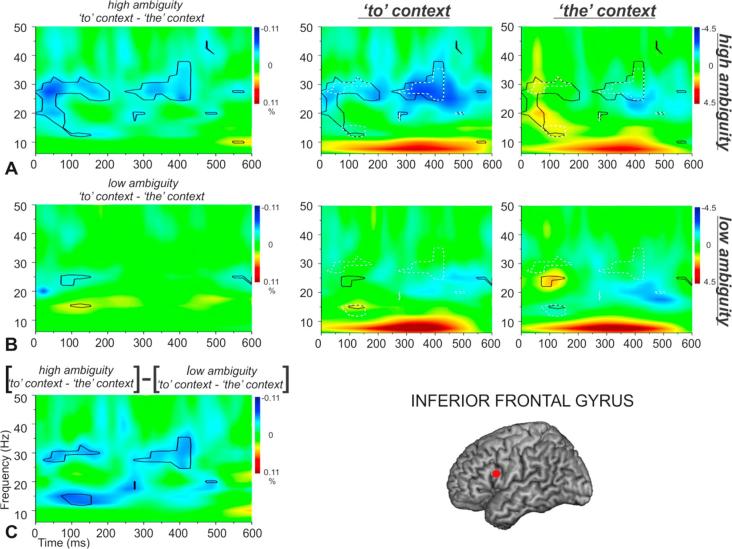
Total power changes and comparisons between conditions for high ambiguity verb phrases (*to* context) and high-ambiguity noun phrases (*the* context) in A, and for low-ambiguity verb phrases (*to* context) and low ambiguous noun phrases (*the* context) in B. Total power changes for each experimental condition are presented in the second and third column, with orange-red and blue-dark blue colours indicating significant power increase or decrease compared to a passive baseline period, respectively. C shows the interaction between Ambiguity and Context in LIFG. In the first column, the black lines enclose regions that are statistically significant in the contrasts between conditions (panel A and B) and in the contrast between A and B (C). In each condition, the between-condition differences are also shown as black lines for the contrasts in A and B, separately, and with white dot-lines for the interaction effects presented in C. (For interpretation of the references to colour in this figure legend, the reader is referred to the web version of this article.)

**Fig. 6:**
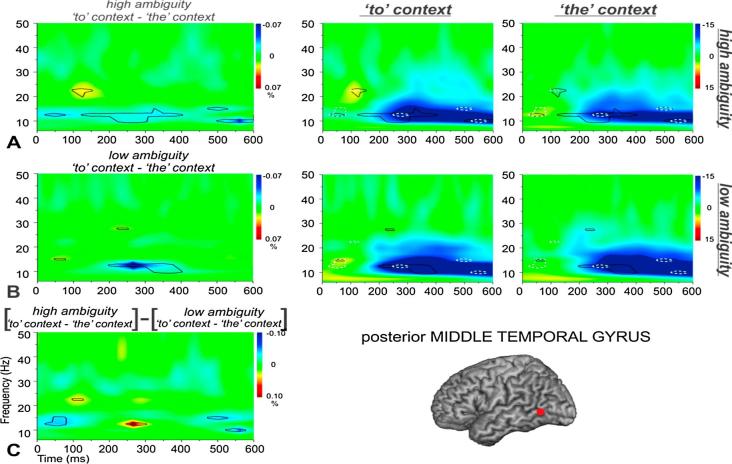
Total power changes and comparisons between conditions for high-ambiguity verb phrases (*to* context) and high-ambiguity noun phrases (*the* context) in A, and for low-ambiguity verb phrases and low-ambiguity noun phrases in B. Total power changes for each experimental condition are presented in the second and third column, with orange-red and blue-dark blue colours indicating significant power increase or decrease compared to a passive baseline period, respectively. C shows the interaction between Ambiguity and Context in LPMTG. In the first column, the black lines enclose regions that are statistically significant in the contrasts between conditions (panels A and B) and in the contrast between A and B (panel C). In each condition, the between-condition differences are also shown as black lines for the contrasts in A and B, separately, and with white dot-lines for the interaction effects presented in C. (For interpretation of the references to colour in this figure legend, the reader is referred to the web version of this article.)

The hypothesis that LIFG and/or LPMTG may play a role in contextually-guided controlled retrieval or subsequent selection of relevant meanings predicts an interaction between ambiguity and context, since the verb > noun context effect should be greater for high-ambiguity words if contextual information is used to resolve ambiguity. To examine this possibility, we compared the effect of context for high-ambiguity words with the effect of context for low-ambiguity words (i.e., the difference of differences) at each site (see Fig. 5, Fig. 6).

Fig. 5 shows the interaction between context and ambiguity for LIFG. Fig. 5A shows the effect of context for high-ambiguity items, Fig. 5B shows the effect of context for low-ambiguity items, and Fig. 5C compares these effects of context across high and low ambiguity items, to confirm if there was an interaction at this site. Fig. 5A shows that high-ambiguity words in verb contexts elicited strong event-related decreases in oscillatory power in LIFG that started within 100 ms of stimulus onset and lasted throughout the epoch. High-ambiguity words in noun contexts showed weaker event-related decreases in oscillatory power (although this response was still seen at 25 Hz and 400 ms post-stimulus onset; around the peak response seen in the verb-context condition), and there was also a transient increase in power at 20 Hz and 50 ms post-stimulus onset for high-ambiguity words in noun contexts. There was a strong difference between these conditions throughout the epoch; i.e., more task-related change in oscillatory power for ambiguous words in verb than noun contexts (plotted in the left-hand column of Fig. 5A). These differences overlapped with the main effects of context in Fig. 3B and ambiguity in Fig. 3A—there were responses to both of these contrasts in LIFG at 150 ms and from 300 to 400 ms, around 25–30 Hz. Thus, it is possible that both of these main effects were driven by the ambiguous verb phrases, and all of these effects reflect the application of context to constrain retrieval, given that verb phrases were the most difficult to process in our pre-test study. Fig. 5B shows that event-related decreases in power were much less marked for low-ambiguity trials in both verb and noun contexts in LIFG, with minimal differences between these conditions. Fig. 5C shows the effect of context was stronger for high-ambiguity than for low-ambiguity items. This pattern of results suggests LIFG might play a significant role in contextually-guided ambiguity resolution.

For LPMTG, the main response was event-related power decreases in the alpha band (8–15 Hz), which were stronger for verb than noun contexts irrespective of ambiguity: this effect of context was seen throughout the epoch for high ambiguity trials (Fig. 6A), and between 200 and 400 ms for low ambiguity trials (Fig. 6B). There were also subtle power *increases* relative to baseline in verb contexts that produced significant differences to noun contexts at around 25 Hz and 100–150 ms for high ambiguity items (Fig. 6A) and 15 Hz and 50–100 ms for low ambiguity items (Fig. 6B). Unlike LIFG, these effects of context in LPMTG did *not* coincide with the effect of ambiguity in time-frequency space. Direct comparisons of the effect of context for high and low ambiguity trials (i.e., the interaction term in Fig. 6C) revealed differences at around 300 ms (10–15 Hz) in the opposite direction to LIFG: i.e., a greater effect of context for the low-ambiguity words, consistent with a role of LPMTG in supplying semantic action features (Fig. 6C). However, there were also regions of time-frequency space that showed a stronger effect of context for high-ambiguity items, at around 100 ms and 20 Hz, plus between 500 and 600 ms, from 10 to 20 Hz. These different interactions over time may occur because, for low-ambiguity words, the context cue and lexical word meaning (action/verb features) agree: therefore, meaning access is easier and earlier, and this is reflected in stronger oscillatory activity for low ambiguity action meanings. In contrast, for high-ambiguity words, interactions between context and lexical word meaning may be needed at various time points in the epoch to guide the selection of relevant features particularly for verbs, and this results in stronger oscillatory activity for high ambiguity action meanings (as for LIFG). These interactions between ambiguity and context suggest that both sites might play a role in contextually guided ambiguity resolution although this pattern is arguably more complex and less striking in LPMTG. As predicted, both sites were sensitive to context at an early stage of processing (within the first 250 ms post-stimulus), consistent with the hypothesis they both support controlled semantic retrieval, by detecting contexts in which retrieval needs to be shaped to suit the circumstances. Both sites also showed the critical interaction later in the epoch, suggesting they might play a role in ambiguity resolution through the contextually-guided selection of relevant semantic information.

## 4 Discussion

The present results help to delineate the temporal dynamics underlying contextually-guided semantic retrieval in LIFG and LPMTG. The findings are broadly consistent with fMRI studies implicating these two regions in (i) tasks in which semantic retrieval is constrained to suit the circumstances (controlled retrieval), (ii) semantic selection between alternative interpretations, and (iii) understanding actions, verbs and events, as opposed to nouns and objects. However, the time and frequency-sensitive nature of MEG allowed us to demonstrate some important similarities between these sites, as follows.

First, as expected, both LIFG and LPMTG showed early interactions between ambiguity and context effects, particularly when comparing high-ambiguity words across nouns and verb contexts. In these cases, the context words (*the* or *to*) provided critical information to bias subsequent semantic retrieval towards the correct action interpretation. These effects were particularly striking for LIFG but both sites showed effects of context earlier than those observed in ERP studies, even before 100 ms post-stimulus. These findings are consistent with the view that LIFG and, to some extent, LPMTG support contextually-guided semantic retrieval. The effects of context might have occurred at such an early stage in this experiment because coarse visual information about the shape of the context word was sufficient to bias subsequent feature retrieval in a useful way. The same context words were repeatedly presented throughout the experiment, and this is likely to have encouraged strategic allocation of attention to relevant features of the stimuli. Consistent with the controlled retrieval view hypothesised in the introduction, searching for, detecting and recognizing verb contexts early on would make phrase interpretation more efficient: in particular, contextual information allows the brain to be configured appropriately to support the later selection of relevant semantic features for ambiguous words according to the context initially established. This view is consistent with the fact that in our pre-test study, ambiguous words in *to* contexts took the longest to processes, whereas *the* contexts were similarly difficult regardless of ambiguity, suggesting that strategic attention to verb contexts may have helped discriminate stimulus types and begun to constrain semantic retreival. These results therefore support a view in which the LIFG and PMTG cooperate in top-down controlled retrieval.

Second, consistent with our predictions, both LIFG and LPMTG showed ambiguity effects around 300 ms and 400 ms post stimulus onset, interaction effects after 300 ms and sustained or recurrent sensitivity to verb contexts from 250 ms onwards. This suggests that both ambiguity and context continued to play a role at this later stage. This is consistent with previous results suggesting that processing demands for morpho-syntactically marked verbs are typically larger than those of noun phrases in LIFG and LPMTG (Tyler et al., 2008, Tyler et al., 2004, Vigliocco et al., 2011), and more generally, with multiple EEG/MEG studies showing semantic integration effects around 400 ms, which have been linked to fronto-temporal interactions in the language network (Federmeier et al., 2000, Kutas and Van Petten, 1994, Wang et al., 2012). Processing demands were also higher for ambiguous words in verb contexts in our behavioural pre-test. Our results therefore suggest that LIFG and LPMTG contributed to the selection of the appropriate interpretation according to the functional context. However, this pattern was again stronger in LIFG: the analysis of LPMTG also showed opposite effects of ambiguity (i.e., greater changes in oscillatory power to low than high ambiguity items) at around 300 ms and 15 Hz.

The findings indicating early sensitivity to context are generally consistent with MEG studies showing early responses (∼100 ms) in posterior temporal cortex to visual word form characteristics as well as to lexical and semantic variables at around 200 ms (Federmeier et al., 2000, Pulvermuller and Shtyrov, 2009, Pulvermüller et al., 2009). The results also cohere with studies showing a rapid response in left frontal cortex to verbs (Pulvermuller & Shtyrov, 2009) and more generally to visually-presented information during word reading (Cornelissen et al., 2009, Klein et al., 2014, Pammer et al., 2004, Pulvermüller et al., 2009). Recent MEG research on sentence processing has also highlighted the predictive nature of sentential contexts and the matching processes that take place between bottom-up and top-down information (Lewis and Bastiaansen, 2015, Lewis et al., 2015). Large-scale functional networks, including the language processing network, are therefore characterised by very rapid and common influences of behaviourally-relevant variables extracted from visual input, which enable these networks to be configured in a suitable way for the efficient extraction of meaning from bottom-up input. When high-ambiguity inputs meet contextual constraints, we would expect the engagement of selection and inhibition mechanisms in LIFG and PMTG, particularly for high-ambiguity words in verb contexts.

Taken together, the present findings have implications for current proposal on LIFG’s functional role. Much fMRI and neuropsychological research has implicated LIFG in at least two aspects of controlled semantic processing (Badre et al., 2005, Davey et al., 2016, Noonan et al., 2013). One view argues that LIFG supports controlled semantic retrieval and mediates inter-regional interactions as a function of task demands via top-down predictions or the establishment of cognitive sets to prepare for upcoming stimulus processing (Badre and Wagner, 2002, Sakai and Passingham, 2006). Additionally, LIFG is proposed to regulate activity in highly competitive situations, where selection or inhibition of competing semantic alternatives is required by the task (Thompson-Schill et al., 1997). The early effects of context and interactions that we observed in LIFG are compatible with a role for this region in establishing an appropriate network for retrieving relevant knowledge. Nevertheless, the processing of an ambiguous phrase is not over at this early stage, as relevant specific semantic features needs to be retrieved. Therefore, the sustained involvement of LIFG for ambiguous phrases may have reflected the selection or inhibition of semantic features relevant to the context, in a process of disambiguation, which was particularly demanding for *to*-contexts. In sum, our results are compatible with the view that mid-LIFG is engaged both by processes that help to constrain on-going retrieval, and by the inhibition of activated knowledge that is irrelevant to the on-going task or context.

Our results also shed light on the role of LPMTG in semantic processing. There remains considerable debate about whether LPMTG is involved in controlled aspects of semantic retrieval (Davey et al., 2015, Davey et al., 2016, Noonan et al., 2013) and/or whether it supports conceptual representation of action knowledge (Kable et al., 2002, Kable et al., 2005, Martin and Chao, 2001). The early and late context and interaction effects found here suggest a role for LPMTG in contextually-guided semantic retrieval of action meanings similar to those of LIFG, in line with controlled retrieval proposals. Thus, LPMTG appears to be engaged when automatic spreading activation of strong features and associations, driven in a bottom-up fashion by the stimulus, is not sufficient for the task and consequently retrieval needs to be constrained to suit the context. However, LPMTG, unlike LIFG, also showed context effects for low-ambiguity items and a reverse interaction after 200 ms onwards, i.e., a stronger response to verb contexts for low-ambiguity items. This finding is consistent with the proposal that this region supports action/event representations. In sum, LPMTG appears to be engaged in processing action semantics features as well as in controlled retrieval at different stages of processing.

We also provide supplementary analysis of LASTG. This site is thought to support semantic processing, particularly in verbal tasks, and in adjective-noun semantic combinations – however, this region has not been implicated in contextually-guided controlled retrieval or in action and verb understanding and it was therefore expected to show a different pattern from LIFG and LPMTG. This expectation was largely confirmed. In particular, LASTG showed a qualitatively different pattern of contextual effects – i.e., stronger task-induced changes for noun than verb contexts. These effects might relate to the stronger responses seen in this site for adjective-noun combinations in previous MEG studies, although unlike those studies, we did not include a non-combination condition (Bemis and Pylkkänen, 2013, Westerlund and Pylkkänen, 2014). In contrast, both LPMTG and LIFG almost exclusively showed stronger responses to verb contexts. LASTG also differed from LIFG in the effects of ambiguity, since LIFG always showed a stronger oscillatory response to ambiguous phrases, while LASTG showed effects of this contrast in both directions. In this way, LASTG and LPMTG were relatively similar – both temporal lobe sites showed responses from 300 to 500 ms that were stronger for low ambiguity items at around 15 Hz, plus stronger responses to more ambiguous items from 400 ms at a higher frequency. Despite this partial similarity between the temporal lobe sites, the analysis provided for LASTG is sufficient to show that there are clear differences across sites for our experimental manipulations, even though all of these sites showed a strong response to the task as a whole within the whole-brain beamforming analysis.

There are some limitations of this study, which should be acknowledged. First, our MEG analysis strategy focussed on the contribution of specific sites – e.g., LIFG and LPMTG – in the processing of context and ambiguity, since these regions are strongly implicated by the fMRI literature and their roles remain controversial. These sites together contribute to a large-scale distributed network potentially including other nodes, but they are not the *only* brain regions supporting this task. Our strategy has been to use whole-brain beamforming to identify sites implicated in the paradigm across conditions in an unbiased way and then to examine differences between conditions using virtual electrodes at specific points-of-interest. This is likely to be a sensitive analysis approach, since previous studies have shown that effects of experimental manipulations tend to be restricted in both time and frequency (Klein et al., 2014, Mollo et al., 2017)– these effects are therefore unlikely to be observed in whole-brain contrasts that aggregate data across broad time-windows or frequency bands. Having localised the effects of interest in time-frequency space, it might be possible for future studies to compute whole-brain contrasts that target these effects. Next, research has suggested that there are functional subdivisions within both LIFG and LPMTG (Badre et al., 2005, Xu et al., 2016). We interrogated local peaks in the whole-brain beamforming data, since MEG is likely to lack the spatial resolution to show distinct response from adjacent regions. Moreover, while the point-of-interest we examined in LIFG was relatively spatially-distinct in our whole-brain beamforming analysis, improving our confidence in the localisation of this point-of-interest, the site in LPMTG was not spatially distinct from the visual response to the task overall. Given the relatively low spatial resolution of MEG, we cannot exclude the possibility that visual signals are contributing to the signals recovered for the LPMTG point-of-interest. Nevertheless, the visual processing demands of the experiment were largely matched across conditions. Thirdly, due to the restricted number of words with balanced frequencies, it was necessary to repeat the stimuli to provide sufficient trials for the analysis. While repetition priming facilitates lexical processing, analysis of the behavioural experiment confirmed that the critical interaction between context and ambiguity was not influenced by this repetition. Nevertheless, further studies are needed to examine the effect of recent experience on the interpretation of balanced ambiguities.

## 5 Conclusions

Our results highlight the intricate dynamics of the engagement of LIFG and LPMTG in semantic retrieval. Both LIFG and LPMTG showed early sensitivity to contextual cues, suggesting they support controlled semantic retrieval by detecting the need to shape retrieval to suit the circumstances, and by maintaining contextually-relevant features. Moreover, both respond until later in the epoch to semantic ambiguity. In LIFG, this effect is consistently stronger for context-dependent action interpretations, suggesting a role in contextually-guided ambiguity resolution. LPMTG shows a similar pattern at discrete points in time (within the first 150 ms and by 500 ms) but this site also showed a stronger response to verb than noun contexts for low-ambiguity items at 250 ms post-stimulus, suggesting a role in processing action meaning. Therefore, different functional roles previously proposed on the basis of fMRI data for LIFG and PMTG are in fact played out at different periods during processing.

## Statement of significance

This work uses MEG to examine the time course of activity in the Inferior Frontal Gyrus and Posterior Temporal Gyrus during context-dependent ambiguity resolution. MEG provides more precise characterizations of the roles of these regions at different stages of processing, which contrast in meaningful ways with those inferred from fMRI.
