# Supplementary material for: Context-dependent lexical ambiguity resolution: MEG evidence for the time-course of activity in left inferior frontal gyrus and posterior middle temporal gyrus
Source: Brain Lang. 2018 Feb-Mar;177-178:23–36. doi: 10.1016/j.bandl.2018.01.001 (PMC5840520; doi:10.1016/j.bandl.2018.01.001)
Supplement: Supplementary data 1 [file mmc1.docx]

**Supplemental Materials**

To examine power changes in the anterior temporal lobe in response to our task and stimuli, we selected a site in anterior superior temporal gyrus (aSTG) as point of interest. The site was located at MNI coordinates *x* = -44, *y* = 24, *z* = -28, and corresponded to a point of peak activation in the whole-brain beamforming analysis in Figure 2 of the main text, driven by power increases between 5-15Hz. Figure SM1 shows the effect of ambiguity in the anterior superior temporal gyrus next to the effects in LIFG already reported in the main text for comparison. While LIFG showed an effect of ambiguity throughout the epoch, the aSTG showed increased power for high-ambiguity phrases much later in processing (~500ms) at 40-50Hz. The aSTG site also showed the opposite effect (greater power change for low-ambiguity phrases, either increases or decreases) at between 200-500ms at 15Hz and between 400-500ms at 20-30Hz. Figure SM2 shows the effect of context for high-ambiguity phrases (e.g., *to bowl* vs. *the bowl*), which were the trials that showed a strong influence of the context manipulation in LIFG. This figure shows that unlike LIFG, the left aSTG showed little sensitivity to the context manipulation, suggesting that it does not play a role in contextually-guided ambiguity resolution, at least when context is specified by the words *to* or *the* in minimal phrases. There was a greater response to noun contexts in aSTG at a late stage (around 550ms at 45Hz), while LIFG showed greater power changes to verb contexts throughout the epoch.

Figure SM1: Total power changes and comparisons between conditions for high and low-ambiguity phrases in A) the left inferior frontal gyrus and B) the anterior superior temporal gyrus. The top panels in A) and B) show total power changes for each ambiguity condition. Orange-red and blue-dark blue colours in the time-frequency plots indicate significant power increase or decrease compared to a passive baseline period, respectively. The lower panels in A) and B) show the differences between the two conditions, with black lines enclosing regions that are statistically significant. These between-condition differences are also shown in the top panels to further qualify the nature of these contrasts. To aid interpretability, crosses and asterisks in the contrast plots represent areas that can be unambiguously attributed to a larger power change relative to baseline in a particular condition (e.g., * = high-ambiguity > low-ambiguity).

Figure SM2: Total power changes and comparisons between context conditions for high-ambiguity phrases in A) the left inferior frontal gyrus and B) the left anterior superior temporal gyrus. The top panels in A) and B) show total power changes for each context condition. Orange-red and blue-dark blue colours in the time-frequency plots indicate significant power increase or decrease compared to a passive baseline period, respectively. The lower panels in A) and B) show the differences between the two conditions, with black lines enclosing regions that are statistically significant. These between-condition differences are also shown in the top panels to further qualify the nature of these contrasts. To aid interpretability, crosses and asterisks in the contrast plots represent areas that can be unambiguously attributed to a larger power change relative to baseline in a particular condition (e.g., * = ‘to’-contexts > ‘the’-contexts).
